# Acquisition Devices for Fetal Phonocardiography: A Scoping Review

**DOI:** 10.3390/bioengineering11040367

**Published:** 2024-04-11

**Authors:** Noemi Giordano, Agnese Sbrollini, Micaela Morettini, Samanta Rosati, Gabriella Balestra, Ennio Gambi, Marco Knaflitz, Laura Burattini

**Affiliations:** 1 Department of Electronics and Telecommunications and PoliToBIOMedLab, Politecnico di Torino, 10129 Torino, Italy; noemi.giordano@polito.it (N.G.); samanta.rosati@polito.it (S.R.); gabriella.balestra@polito.it (G.B.); marco.knaflitz@polito.it (M.K.); 2 Department of Information Engineering, Engineering Faculty, Università Politecnica delle Marche, 60131 Ancona, Italy; a.sbrollini@staff.univpm.it (A.S.); m.morettini@univpm.it (M.M.); e.gambi@univpm.it (E.G.)

**Keywords:** phonocardiography, fetal heart sounds, fetal monitoring, pregnancy, research prototypes, commercial devices, multi-channel recording, single-channel phonocardiography, multimodality

## Abstract

Timely and reliable fetal monitoring is crucial to prevent adverse events during pregnancy and delivery. Fetal phonocardiography, i.e., the recording of fetal heart sounds, is emerging as a novel possibility to monitor fetal health status. Indeed, due to its passive nature and its noninvasiveness, the technique is suitable for long-term monitoring and for telemonitoring applications. Despite the high share of literature focusing on signal processing, no previous work has reviewed the technological hardware solutions devoted to the recording of fetal heart sounds. Thus, the aim of this scoping review is to collect information regarding the acquisition devices for fetal phonocardiography (FPCG), focusing on technical specifications and clinical use. Overall, PRISMA-guidelines-based analysis selected 57 studies that described 26 research prototypes and eight commercial devices for FPCG acquisition. Results of our review study reveal that no commercial devices were designed for fetal-specific purposes, that the latest advances involve the use of multiple microphones and sensors, and that no quantitative validation was usually performed. By highlighting the past and future trends and the most relevant innovations from both a technical and clinical perspective, this review will represent a useful reference for the evaluation of different acquisition devices and for the development of new FPCG-based systems for fetal monitoring.

## 1. Introduction

According to the World Health Organization (WHO) mortality database [1], in 2023 perinatal deaths were 11,917, which represents 2% of all births. The main complications may be related to fetal diseases, such are hypoxia or intrauterine growth retardation, or to maternal diseases, such are preeclampsia or gestational diabetes [2,3]. The prevention of adverse events during pregnancy and delivery is provided by prenatal monitoring, which represents a crucial step for the maternal and fetal health status assessment. Clinical guidelines suggest periodical screening, usually composed of blood and urine tests, ultrasound imaging screening, and, if suggested by complications, genetic tests [4]. Moreover, in the last phases of the pregnancy, Fetal Heart Rate (FHR) is registered weekly in combination with the maternal uterine contractions. Due to the high costs, this practice cannot be performed for a long time, causing possible missing identification of adverse events. Nowadays, portable and wearable technologies have spread across the healthcare market, opening to the possibility of having devices able to perform a long continuous recording [5]. Considering this innovation, a new wearable home-monitoring device would be an optimal solution for continuous prenatal monitoring. The ideal system should be designed to be noninvasive for both mother and fetus (also for long periods), independent of any clinical personnel, and user-friendly for the subject.

**Figure 1:**
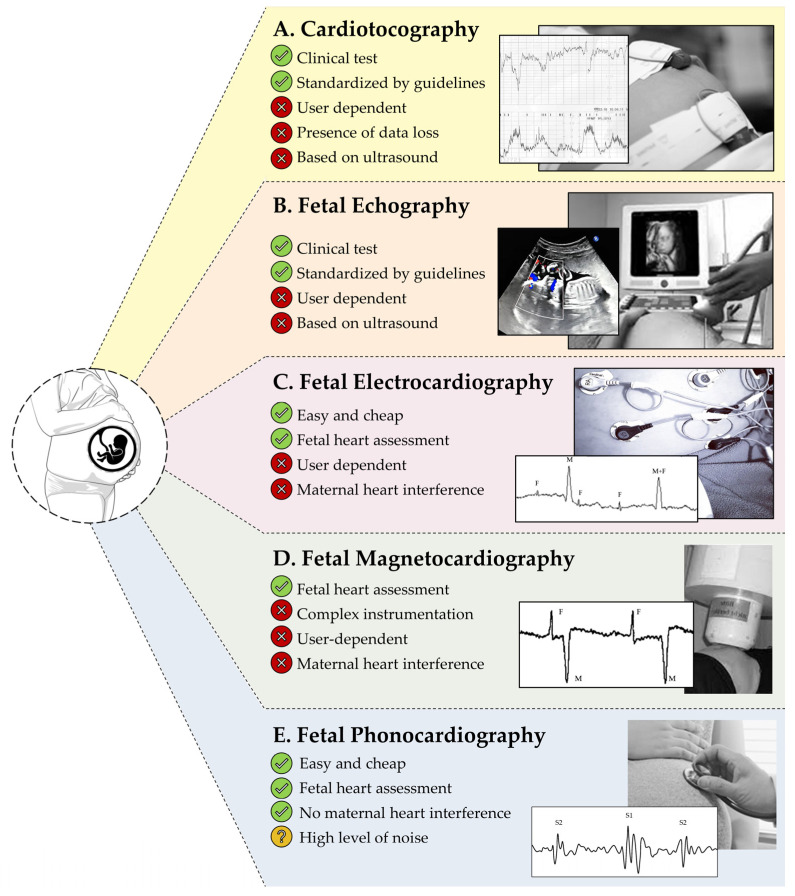
Five used screening tests for fetal monitoring: (**A**) cardiotocography, (**B**) fetal echography, (**C**) fetal electrocardiography, (**D**) fetal magnetocardiography, (**E**) fetal phonocardiography.

In the literature, there are five screening tests for fetal monitoring (Figure 1). The routine clinical method for fetal wellbeing assessment is the measurement of FHR, called Electronic Fetal Monitoring (EFM) and introduced in the 1960s [6]. Afterwards, clinical organizations, such as the International Federation of Gynecology and Obstetrics (FIGO), the American College of Obstetricians and Gynecologists (ACOG), and the National Institute of Clinical Excellence (NICE), recognized the utility of combining EFM with maternal Uterine Contractions (UC) to optimize the outcomes for both mother and infant [7,8,9,10]. This combined simultaneous recording of FHR and UC is nowadays known as cardiotocography (CTG; Figure 1A) [11].

Another clinical technology for fetal health status assessment is the fetal echography (FECHO; Figure 1B), which consists of using ultrasound signals for the reconstruction of the images of the fetal body structures. Periodically performed during the pregnancy, FECHO images can be manually annotated by the clinical personnel, who assess the fetal health status and growth by measuring some anatomical biometry index, such as the head circumference, the abdominal circumference, and the femur length [12,13]. CTG and FECHO are widely diffused and accepted in clinical practice, but their probes are highly sensitive to noise, requiring frequent repositioning of the sensors by clinical personnel and, thus, being user-dependent [8,11,12,13]. Moreover, both of these technologies seem not to be suitable for long recording, considering the recent evidence of potential harmful effects of sustained ultrasonic radiation on the fetus [11]. These disadvantages make CTG and FECHO not suitable for the realization of a new home-monitoring device.

In the literature, noninvasive fetal electrocardiography (FECG; Figure 1C) and fetal magnetocardiography (FMCG; Figure 1D) were presented as innovative techniques back in the early 2000s [14,15]. Specifically, FECG consists of noninvasive recording of the fetal electrocardiogram by applying multiple electrodes to the maternal abdomen [14]. Instead, FMCG consists of noninvasively measuring the magnetic field of the fetal heart, using SQUID sensors placed over the maternal abdomen [15]. While FECG acquisition setting is very simple and cheap for the pregnant woman, FMCG acquisition requires complex and expensive instrumentation. Moreover, both FECG and FMCG require important modifications by signal processing techniques to remove the high level of noise, especially due to maternal heart interference [16], which is a highly challenging interference considering its frequency-band superimposition with fetal heart activity [14]. Finally, both FECG and FMCG are user-dependent because the electrode and probe location are standardized according to the maternal abdomen and not to fetal position; thus, this technology requires support by clinical personnel for the identification of the best sensor location for the recording of fetal heart activity [14]. Thus, also in these cases, considering their technical limitations, FECG and FMCG seem not to be suitable for fetal home-monitoring.

In this context, fetal phonocardiography (FPCG; Figure 1E) represents an easy, cheap, and noninvasive alternative technique. Discovered in the 1750s by Kergaradec, Marsac, and Kennedy via Pinard’s stethoscope as qualitative auscultation [17], FPCG consists in the recording of the fetal heart sounds generated by the mechanical activity of the heart: ventricular heart contraction provokes the closure of the mitral and tricuspid valves, whose sound is called first heart sound, and the ventricular heart relaxation provokes the closure of the pulmonary and aortic valves, whose sound is called second heart sound. Timing between these sounds directly measures heart systole and diastole and can be used for FHR assessment [18]. Nowadays, FPCG is used for FHR auscultation during the CTG test, and it has never been used for clinical diagnosis. The main reason relates to the high level of interferences, provoked by amniotic fluid, digestive activity, and fetal movements [11]. Despite the high level of noise, maternal heart activity interference is not a high corruption factor, and interferences are mainly located in frequency bands different from that of interest. Thus, FPCG is not based on ultrasound technologies and its denoising is more viable. Therefore, FPCG seems to be the optimal candidate for the development of a new wearable home-monitoring device for continuous prenatal monitoring, considering the high advantages in comparison with the other technologies for fetal monitoring.

In the literature, two reviews focused on the FPCG recording for fetal wellbeing assessment [19,20] by considering FPCG physiological aspects. Kovács et al. [19] presented an overview of the development of FPCG, emphasizing its successful applicability for long-term fetal measurements and home monitoring. Adithya et al. [20] mainly focused on the signal processing procedures for FPCG analysis, emphasizing the feature of interest, the main useful denoising procedure, and the open research challenges. None of these systematic reviews investigated the technologies and system innovations in the field of FPCG acquisition. Thus, the present review investigates the innovative research prototypes and commercial devices for the acquisition of phonocardiogram in the field of fetal monitoring with the aim to define the trends in FPCG device use and to identify the main challenges that should be solved before they can be considered suitable for home-monitoring. Thus, in the field of fetal phonocardiography, the main contributions of the present review paper are the following: (1) the collection of innovative studies related to the use of research prototypes and commercial devices, (2) a description of the novel trends in the field, (3) an indication of the technical and clinical validation of a novel device, and (4) the collection of the still open challenges.

## 2. Materials and Methods

The literature search, screening, and analysis were conducted in adherence to the PRISMA extension for scoping reviews (PRISMA-ScR) [21].

### 2.1. Literature Search Strategy

The literature search was conducted on three electronic databases—Scopus, PubMed, and Web of Science—in order to guarantee the presence of peer-reviewed studies. The search query was constructed by combining three concepts:

- Eligible works must be related to fetal monitoring. Identified keywords: “fetal”, “pregnancy”, “fetus”, “prenatal”, “antenatal”, “foetal”, “foetus”;
- Eligible works must be related to heart sounds. Identified keywords: “phonocardiography”, “heart sounds”, “FPCG”, “PCG”, “heart murmur”, “acoustic cardiography”, “auscultation”;
- Eligible works must provide technical information concerning the device design. Identified keywords: “hardware”, “device”, “system”, “recording”, “acquisition”, “microphone”.

Each keyword related to the same concept was combined using a Boolean operator “OR”. The three sets of keywords were combined using the Boolean operator “AND”.

Details about the search parameters for each database are reported in Table 1 to ensure the repeatability of the search. The choice of the search parameters was driven by the databases’ options as well as the need for obtaining the highest consistency possible No constraint was applied concerning the time range to explore the evolution of the technology from its birth to date. No constraint was applied concerning the quality of the bibliometric source.

### 2.2. Selection of Studies

Before starting the screening process, duplicate records were automatically removed along with the following types of records:

- Written in a language other than English;
- Belonging to one of the following document types: guidelines, conference collection, and editorial.

The screening process accounted for three phases:

1. Analysis of the title;
2. Analysis of the abstract (if available);
3. Analysis of the full text (if available).

The same exclusion criteria, detailed below, were used in all three phases (title, abstract and full-text analysis, respectively). Review articles were excluded in the last phase. The exclusion criteria were defined by considering that all the above-mentioned concepts (technical description of the device, technology based on heart sounds, and monitoring of the fetus) must be present to make the study includable. Therefore, the following exclusion criteria were applied:

- Non-technical studies (i.e., studies using fetal auscultation for clinical purposes, without focusing on the device) were excluded;
- Studies describing devices not supporting the recording of heart sounds were excluded;
- Studies describing devices for the recording of heart sounds in children or adults were excluded;
- Studies focusing on signal processing, not providing a description of the device, were excluded.

During the third phase, i.e., the analysis of the full text, citation searching was carried out by screening the reference list of the included studies. Studies meeting the inclusion criteria of the review were added. Among those, studies without an available full text were excluded. Studies from the search of the databases and studies from the citation search were analyzed together in the following steps.

### 2.3. Data Charting and Synthesis

All included studies were analyzed to assess the following features:

- **Device**: number, type and position of microphone sensors, availability of other types of sensors, type of sensor–skin interface (head of the sensor), fasten means to the maternal abdomen, system architecture, hardware characteristics (filters, gain, ADC dynamics, sampling frequency, type of processor, memory, power supply);
- **Signal processing**: method for denoising, method for FHR estimation, other processing;
- **Validation**: goal of the validation study, comparison against a gold standard, size of the sample population, gestational age, performances.

Data were synthesized in tables. Studies using the same device were grouped together, and the hardware characteristics were merged. The features related to signal processing and validation were kept separated.

For the analysis, studies were first divided into two groups depending on whether they employed novel research prototypes (RPs) or commercial devices (CDs). For RPs, all the above-mentioned device features were charted. For CDs, the hardware characteristics were typically not mentioned either in the paper or in the device user manual and were not charted. The following features were added for CDs:

- Manufacturer and commercial name;
- Availability on the market at the search date;
- CE mark according to the Medical Device Regulation 2017/745;
- Whether the device is specifically designed for fetal application or not.

**Figure 2:**
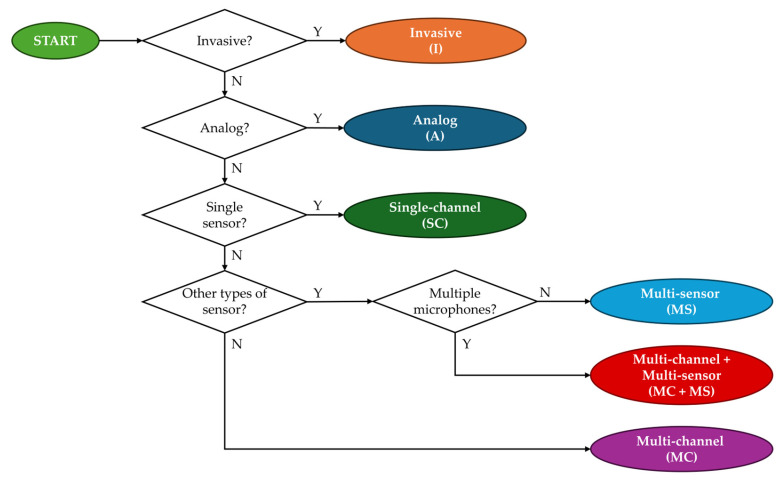
Classification algorithm of the results of the literature search for the analysis.

For each of the two groups (RP and CD), devices were divided into classes according to the device’s features. The classification algorithm is graphically described in Figure 2.

The data synthesis was performed according to the obtained results. For each feature, the results were manually divided into relevant categories, and, when possible, the distribution of each category was mapped, both in general terms and in function of time. For complex features, such as the type of the sensor–skin interface (head of the sensor) and the system architecture, some templates were defined based on the results; devices were grouped according to the defined templates. For numerical features, the range and the median of the results were computed. Features with a high number of missing values were not further analyzed.

## 3. Results

This section presents the results of the scoping review. First, the results of the literature search phase will be presented in the PRISMA-ScR format. Then, the devices proposed in the included studies will be classified (classification algorithms are proposed in Figure 2). In the end, the results of the data charting and analysis phase will be presented. Hardware features for research prototypes and commercial devices, respectively, will also be presented, along with the signal processing and validation features found in the included studies.

### 3.1. Literature Search

**Figure 3:**
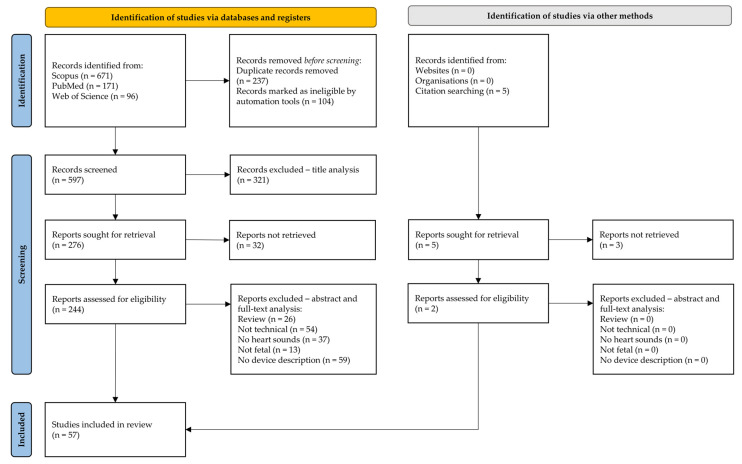
Flowchart of the literature search (identification, screening, and inclusion phases) in complete adherence to PRISMA extension for scoping reviews (PRISMA-ScR).

The result of the literature search in the databases produced 671 studies from Scopus, 171 studies from PubMed, and 96 studies from Web of Science, having overall 938 studies. Figure 3 summarizes the procedure of the systematic literature search selection. Finally, 57 studies were included in the review.

**Figure 4:**
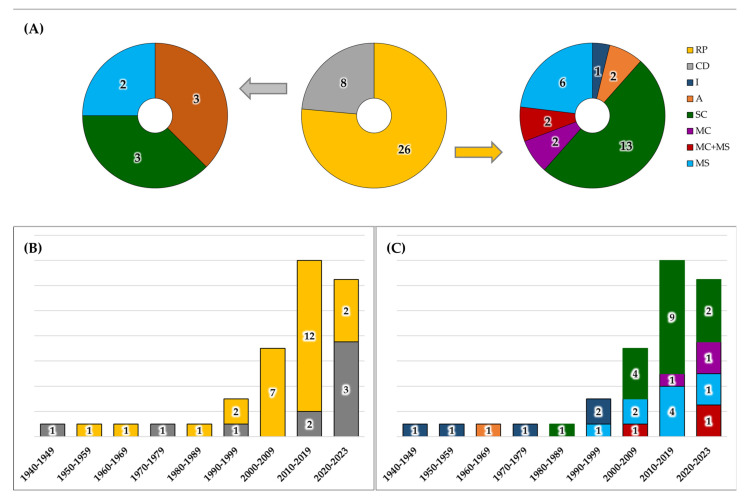
Device classification in research prototypes (RPs) and commercial devices (CDs), and stratification in analog (A), invasive (I), single-channel (SC), multi-channel (MC), and multi-sensory (MS) (Panel (**A**)) and their trends over time (Panel (**B**) and Panel (**C**)).

The publication year of selected studies ranges from 1941 to 2023, and they can be classified into 31 journal papers, 25 conference contributions, and one book chapter. The studies described 34 devices for FPCG acquisition, divided into 26 RPs and 8 CDs (Figure 4A). Among the RPs, one belongs to I class, two belong to A class, thirteen belong to SC class, two belong to MC class, six belong to MS class, and two belong to both MC and MS classes. Among CDs, three belong to A class, three belong to SC class, and two belong to MS class. The analysis reveals that in the field of device design and development, research typically spans over periods longer than 5 years, because it has to include the phases of design, implementation, prototyping, bench testing, and clinical validation. Figure 4B,C summarize the development of devices, classifying them according to the RP and CD classification and type classes, respectively. In both graphs, the size of the bars was normalized according to the number of years in the series class, while the label represents the number of devices in each specific class. 

### 3.2. Hardware Characteristics

**Figure 5:**
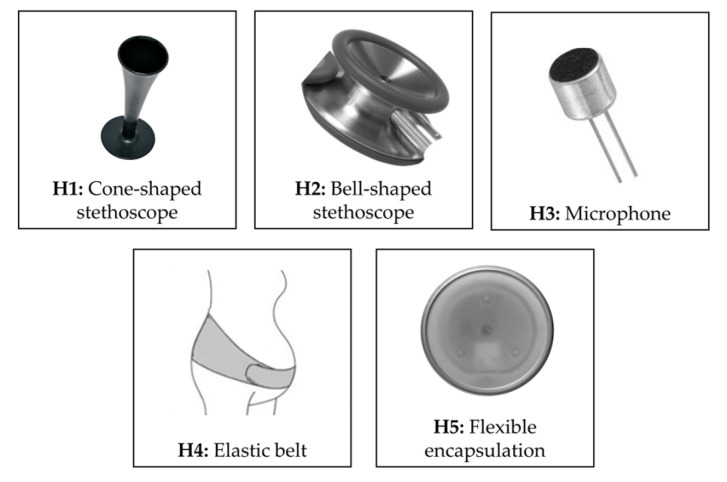
Type of sensor–skin interface (i.e., head of the sensors) reported in the included studies.

**Figure 6:**
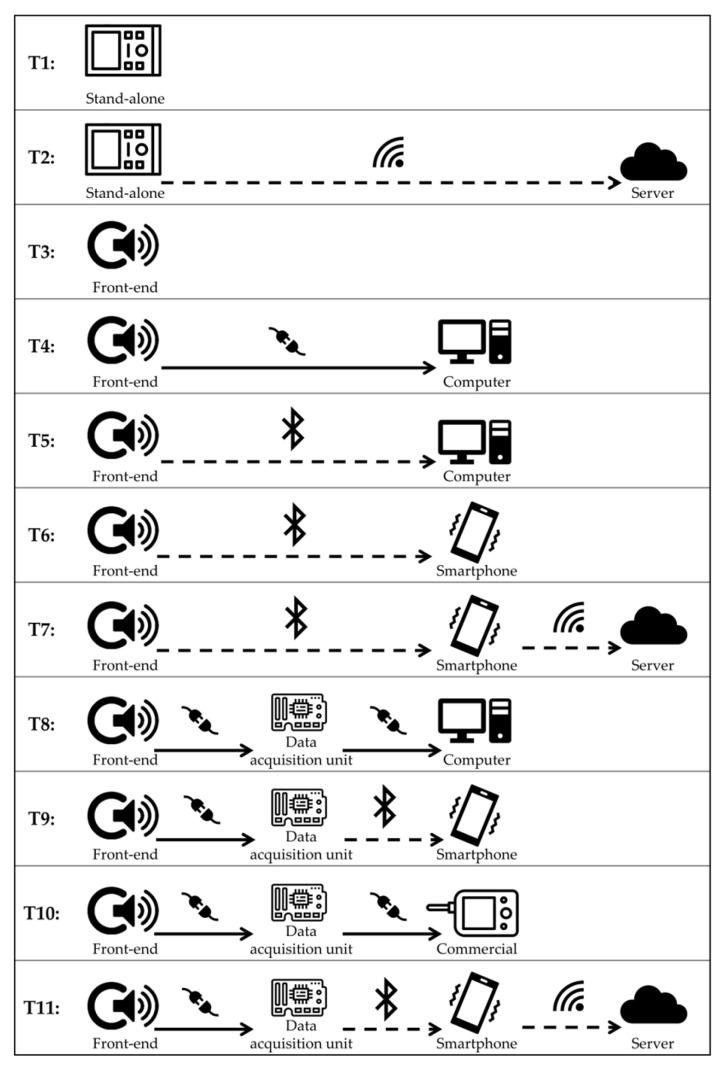
Templates of the system architecture reported in the included studies.

Table 2 and Table 3 summarize the hardware features in terms of sensors, system, and hardware characteristics of RPs and CDs, respectively. Results of the studies revealed different types of architecture systems and different types of sensors associated with the devices. According to this finding, we stratified the devices according to five types of head of the sensors (H1–H5), schematically represented in Figure 5, and 11 architecture system templates (T1–T11), schematically represented in Figure 6.

### 3.3. Signal Processing and Clinical Validation

Table 4 summarizes the signal processing features and clinical validation procedures of the studies that involved RPs and CDs.

**Table 2:** Data charting of the studies describing new research prototypes (RPs). Hardware features.

Study			Sensors				System			Hardware Characteristics						
Dev./ Ref.	Year	Class	N	Type	Position	Other	Head Type	Arch.	Fasten Means	Filters	Gain	ADC	SF	Processor	Memory	Power Supply
**RP1**/ [22,23]	1993	A	3–7	Piezo-electric	Depends on fetus position	-	H4	T1	Belt	20 Hz–55 Hz	Y	-	-	-	-	-
**RP2**/ [24]	1953	A	1	Piezo-electric	-	-	H2	T1	-	-	>10^5^	-	-	-	-	-
**RP3**/ [25]	1964	I	1	Sonic transd.	Intrauterine	-	-	T1	-	-	Y	-	-	-	-	-
**RP4**/ [26]	2019	SC	1	Electret	-	-	H1	T7	-	N	N	N	16 kHz	N	N	Via phone
**RP5**/ [27]	2019	SC	1	Electret	Depends on fetus position	-	H1	T6	Adhesive tape	-	-	16 bit	44.1 kHz	-	-	-
**RP6**/ [28]	2019	SC	1	Electret	-	-	H3	T9	-	15 Hz–3 kHz	9	16 bit	48 kHz	CSR8670/master control chip	16 Mb flash memory	-
**RP7**/ [29,30,31]	2018	SC	1–3	Electret	Depends on fetus position	-	H1 H2	T8	Handheld	16 Hz–20 kHz	Y	16 bit	5 kHz	ARM Cortex-M4	-	Battery
**RP8**/ [32,33]	2018	SC	1	Piezo-electric	-	-	H2	T9	-	-	12	-	44.1 kHz	-	-	Battery
**RP9**/ [34]	2017	SC	1	Electret	-	-	H3	T10	-	DC to 200 Hz	Y	-	-	Arduino Uno R3 (ATMega328)	-	Battery
**RP10**/ [35]	2017	SC	1	-	3 cm left/right, 1 cm up/down the navel	-	H2	T9	-	-	Y	-	-	-	-	-
**RP11**/ [36]	2012	SC	1	Condenser	-	-	H1	T11	-	-	Y	-	-	-	-	-
**RP12**/ [37,38]	2008	SC	1	Piezo-electric	-	-	H1	T8	-	DC to 70 Hz	-	-	8 kHz	-	-	-
**RP13**/ [39,40]	2006	SC	1	Electret	Beside CTG	-	H1	T10	-	DC to 110 Hz	Adj.	16 bit	2 kHz	Intel XScale 624 MHz	128 MB Flash ROM + 64 MB SDRAM	Battery
**RP14**/ [41]	2003	SC	1	-	-	-	H3	T8	-	-	-	16 bit	1 kHz	-	-	-
**RP15**/ [42]	2000	SC	1	-	-	-	H2	T5	-	-	-	-	-	-	-	-
**RP16**/ [43,44,45,46,47]	1986	SC	1	Piezo-electric (TAPHO sensor)	Depends on fetus position	-	H3	T3	Double-sided adhesive discs	-	-	-	-	-	-	-
**RP17**/ [48]	2020	MC	3	MEMS	100 mm triangle	-	H5	T7	-	-	Y	16 bit	5 kHz	ARM Micro Control Unit	-	-
**RP18**/ [11,49]	2017	MC	4	Piezo-electric	160 mm square around the navel	-	H5	T8	3D-printed harness	-	-	16 bit	1 kHz	-	-	-
**RP19**/ [50]	2023	MC + MS	3	Condenser	Depends on fetus position	3 electrodes for EHG recording	H4	T9	Elastic belt	-	-	32 bit	-	Shakti Parashu processor (Arty A7-100T FPGA)	128 MB RAM	Battery
**RP20**/ [51,52]	2000	MC + MS	6	Sound guide	-	Microphone for mPCG	H3	T8	Strap	DC to 200 Hz	1 to 8	12 bit	1024 Hz	-	-	-
**RP21**/ [53,54]	2018	MS	1	Piezo-electric	-	Microphone for mPCG	H3	T6	-	DC to 80 Hz	AGC	-	1 kHz	-	Y	Battery
**RP22**/ [55,56,57,58,59]	2017	MS	1	Fiber optic	-	Microphone for mPCG	H5	T8	Self-adhesive straps	0.5 Hz–400 Hz	1 to 50	16 bit	1 kHz	NI USB 6210 card	-	-
**RP23**/ [60,61]	2013	MS	1	-	-	Microphone for ambient noise	H2	T7	-	-	Adj.	24 bit	2 kHz	MSP430 microcontroller by TI	32 GB flash mem.	Battery
**RP24**/ [62]	2007	MS	1	Piezo-electric	-	Microphone for ambient noise	H1	T8	-	DC to 70 Hz	Y	-	-	-	-	Battery
**RP25**/ [63,64]	2001	MS	1	Electret	-	Microphone for ambient noise	H1	T4	Handheld	-	Y	16 bit	11,025 Hz	-	-	Battery
**RP26**/ [65,66]	1991	MS	1	Inductive (INPHO)	Few cm below the navel	Electrode for FECG/mECG	H3	T4	Double-sided tape	DC to 200 Hz	Y	12 bit	640 Hz	Olivetti M290 microcomputer	20 MB	-

ADC: analog-to-digital converter; Arch.: architecture; DC: direct current; Dev.: device; EHG: electrohysterography; N: no; Ref.: reference; ROM: read-only memory; Transd.: transducer; Y: yes.

**Table 3:** Data charting of the studies involving the validation of a commercial device (CD) for fetal use. Hardware features.

Study			Commercial Information				Sensors				System		
Dev./ Ref.	Year	Class	Manufacturer	Commercial Name	On Market	CE Mark	N	Type	Position	Other	Head Type	Arch.	Fasten Means
**CD1**/ [67]	1941	A	Cambridge Instrument Inc. (London, UK)	Electrocardiograph-stethograph	N	-	1	-	Depends on fetus position	Electrodes for FECG	-	T1	Rubber strap
**CD2**/ [68]	1970	A	Jaeger Laboratories (Columbus, OH, USA)	Model 3 solid-state amplifier	N	-	6	Generic mic.	-	-	-	T1	Rubber strap
**CD3**/ [69]	1993	A	Smith Kline Instruments (Sunnyvale, CA, USA)	Smith Kline Model EKS-1	N	-	1	-	-	-	-	-	-
**CD4**/ [70]	2020	SC	Ayu Devices (Mumbai, India)	Ayusynk digital stethoscope	Y	N	1	-	-	N	H2	T3	Handheld
**CD5**/ [70]	2020	SC	TE Connectivity (Schaffausen, Switzerland)	Contact microphone CM-01b	Y	N	1	Piezo-electric	-	-	H3	T3	-
**CD6**/ [71]	2015	SC	GS Technology (Seoul, Republic of Korea)	JABES	Y	Y	1	-	Lower abdomen, follows analog auscultation	N	H2	T4	Handheld
**CD7**/ [70,72,73]	2022	MS	BIOPAC Systems Inc. (Goleta, CA, USA)	MP36 system + SS30LA/SS30L	Y	Y	1	Piezo-electric	-	Electrodes/microphones can be added	H2	T4	Handheld
**CD8**/ [74,75,76,77]	2018	MS	ADInstruments (Sydney, NSW, Australia)	Cardio Microphone MLT201 + Powerlab acquisition system	Y	Y	1	Electret	-	Electrodes/microphones can be added	H2	T8	Adhesive tape

Arch.: architecture; Dev.: device; N: no; Ref.: reference; Y: yes.

**Table 4:** Data charting of all the included studies. Signal processing and validation features.

Study				Signal Processing			Validation				
Dev	Ref	Year	Class	Denoising	FHR Estimation	Other	Goal	Gold Standard	Pop. Size	Gest. Age (Weeks)	Results
**RP1**	[22]	1990	A	-	-	Power spectrum density	Sound	-	-	35–39	Qualitative appraisal
	[23]	1993		-	Y	Heart sound detection (linear prediction)	Sound	CTG	16	-	In vitro: calibration error = ±1.3 dB to ±2.5 dB. In vivo: qualitative appraisal.
**RP2**	[24]	1953	A	-	-	-	Sound	-	-	-	Qualitative appraisal
**RP3**	[25]	1964	I	-	-	-	-	-	-	-	-
**RP4**	[26]	2019	SC	Butterworth BPF 20–200 Hz + WT	Cyclostationary process in frequency domain	-	FHR	Doppler	10	36–39	Agreement = 96% (Bland–Altman)
**RP5**	[27]	2019	SC	-	-	-	Sound	Electronic steth.	5	>37	Increased SNR, decreased loss due to artifacts
**RP6**	[28]	2019	SC	-	-	Play the sound	Sound	-	-	-	-
**RP7**	[29]	2018	SC	-	Y	-	FHR	CTG	1	-	Qualitative appraisal
	[30]	2018		BPF 0.5–70 Hz	Envelope-based	-	FHR	CTG	1	34	Measured FHR = 133 bpm vs. CTG = 120–150 bpm
	[31]	2019		FIR BPF 20–110 Hz	-	PSD	Sound PSD	-	1	34	Qualitative appraisal
**RP8**	[32]	2018	SC	BPF 10–400 Hz + SVR, EMD, adaptive LMS, Wavelet	-	-	Sound	-	-	-	SVR best denoising; qualitative appraisal
	[33]	2021		SVR, EMD, adaptive LMS, WT	Time windowing	-	FHR	-	-	-	SVR best denoising; qualitative appraisal
**RP9**	[34]	2017	SC	-	Peak detection	-	FHR	Electronic stethoscope	10	13–38	Bias = −1.2% to 1.4%, Tolerance = ±5 bpm
**RP10**	[35]	2017	SC	-	Y	-	FHR	-	7	-	Separation FPCG/MPCG possible in 70% of cases
**RP11**	[36]	2012	SC	DWT	Envelope-based	-	FHR	Doppler	15	28–38	Acc = 98%
**RP12**	[37]	2008	SC	FIR BPF 20–200 Hz	Manual labelling S1	-	Sound FHR	Doppler	21	36–40	Clear sound in 15/21 cases, Acc = 98%
	[38]	2011		DWT	-	Heart sounds segmentation (envelope-based) + CWT	Time frequency	-	18	-	Qualitative appraisal
**RP13**	[39,40]	2006	SC	Butterworth IIR HPF 35 Hz	Envelope-based	Confidence factor estimation	FHR	CTG	41	37–38	Agreement = 75%; qualitative appraisal
**RP14**	[41]	2003	SC	FIR BPF 10–50 Hz	Envelope-based	Heart sounds detection	FHR	Monitor	2	-	-
**RP15**	[42]	2000	SC	-	Adaptive correlation	-	FHR	-	10	-	Qualitative appraisal
**RP16**	[43]	1986	SC	HPF 50 Hz	-	-	Sound	-	140	20–29	Heart sounds detection; on qualitative appraisal: 0% in <20 w, 22% in 20–24 w, 83% in 25–29 w, 100% in >30 w
	[44]	1986		HPF 50 Hz	-	-	-	-	-	-	-
	[45]	1986		HPF 40 Hz	-	Manual labelling	Systolic time vs. breathing/moving	FECHO	12	28–41	R = 0.86 Systolic time vs. fetal breathing
	[46]	1989		BPF 45 Hz to 65 Hz	Full-wave rectifier + variable comb filtering	-	FHR	Scalp FECG	-	-	AE < 3%
	[47]	1989		HPF 20 Hz	-	Heart sounds segmentation (peak detection)	Systolic time	-	-	-	-
**RP17**	[48]	2020	MC	BPF (not spec.) + DWT	Y (not specified)	Fetal heart localization (CNN on power images)	FHR fetal position	Monitor, B-scan US	16	-	FHR:AE = 4.3 bpm, Fetal location: Acc = 100% (tolerance 33 mm)
**RP18**	[11]	2017	MC	Notch filter 50 Hz	Peak detection	FPCG/mPCG separation (BSS)	FHR	CTG	20	-	AE = −0.21 bpm (2SD = ±3 bpm)
	[49]	2018		WTST-NST	Peak detection	FPCG/mPCG/mResp separation (refBSS)	FHR	FECG, Doppler	15	33–40	R = 0.96 vs. FECG
**RP19**	[50]	2023	MC + MS	-	Y	-	FHR	Doppler	10	28–40	Qualitative appraisal
**RP20**	[51]	2000	MC + MS	LPF 188.1 Hz	Y	-	FHR	-	1	37	Qualitative appraisal
	[52]	2003		LPF 188.1 Hz	-	Power spectrum analysis	Sound PSD	-	1	37	Qualitative appraisal
**RP21**	[53]	2018	MS	-	Y	-	FHR	Monitor	50	-	AE ≤ 2 bpm
	[54]	2018		BPF	Envelope-based	-	FHR	Monitor	50	-	AE ≤ 2 bpm, agreement on fetal heart status = 100%
**RP22**	[55]	2017	MS	-	Y	FPCG/mPCG separation (adaptive + nLMS)	FHR Sound SNR	-	8	36–42	Qualitative appraisal
	[56]	2017		-	-	FPCG/mPCG separation (adaptive + nLMS)	Sound SNR	-	-	-	Tested on simulated data
	[57]	2017		-	-	FPCG/mPCG separation (RLS)	Sound SNR	-	5	-	Qualitative appraisal
	[58]	2017		-	Peak detection	FPCG/mPCG separation (adaptive + LMS/nLMS)	FHR Sound SNR	-	10	35–42	Qualitative appraisal
	[59]	2018		-	-	FPCG/mPCG separation (LMS/RLS)	Sound SNR	-	-	-	Tested on simulated data
**RP23**	[60]	2013	MS	-	-	-	-	-	-	-	-
	[61]	2014		Computational auditory scene analysis	Adaptive matching	-	FHR	Doppler	8	37–40	AE < 10%
**RP24**	[62]	2007	MS	Adaptive filter	Y	-	FHR	CTG	16	36–40	Acc = 97.95%
**RP25**	[63]	2001	MS	DWT + BPF 35–200 Hz + adaptive cross-channel cancellation	-	-	Sound	-	3	-	Qualitative appraisal
	[64]	2003		BPF 35–200 Hz + DWT	Envelope + cross-correlation	-	FHR	CTG	9	28–40	2:Acc > 90%; 3:Acc > 80%; 2:Acc > 70%; 2:Acc < 70%
**RP26**	[65]	1991	MS	Adaptive filter	-	-	SNR	-	5	-	Qualitative appraisal
	[66]	1991		BPF 40–80 Hz	Y	Fetal movement identification	FHR, fetal moving	FECHO, IUP	6	36–39	SNR = 96 dB in lab SNR = 78 dB in real data
**CD1**	[67]	1941	A	-	-	Manual labelling	Alive fetus	-	40	32–40	Acc = 100%
**CD2**	[68]	1970	MC	-	-	-	Sound	-	-	-	-
**CD3**	[69]	1993	SC	-	-	-	-	-	-	-	-
**CD4**	[70]	2020	SC	-	-	-	SNR	-	-	-	-
**CD5**	[70]	2020	SC	-	-	-	SNR	-	-	-	-
**CD6**	[71]	2015	SC	-	Y	Single-channel BSS (EMD + NNMF)	FHR	FECHO	50	30–40	Acc = 96%
**CD7**	[72]	2001	MS	-	Multiresolution analysis + Hilbert transform	Estimation of the HRV signal and extraction of variability indexes	FHR and its analysis	FECG	11	28–41	MS2 was found as the only reliable time reference
	[70]	2020		-	-	-	SNR	-	-	-	-
	[73]	2022		BPF (adjustable frequencies)	Envelope + cross-correlation	-	FHR	Manual	99	30–40	Manual annotation tested on public dataset with AE = 0.85 bpm. In vivo, 60/99 recordings with sufficient quality, MAE = 7.54, PPV = 87%
**CD8**	[74]	2018	MS	BPF 20–250 Hz	Envelope-based	-	FHR	CTG	9	24–39	R = 0.64 to 0.84
	[75]	2019		BPF 20–200 Hz	Spectrogram + NNMF	-	FHR	CTG	4	38–39	R ≈ 0.9
	[76]	2022		BPF 20–250 Hz	Spectrogram + NNMF	-	FHR	CTG	38	>37	25/38 suitable recordings; qualitative appraisal; average agreement = 75%
	[77]	2023		FIR BPF 20–200 Hz	Spectrogram + NNMF + HMM + modified Viterbi algorithm	-	FHR	CTG	6	38–39	Modified Viterbi algorithm reduces confusion with maternal HR to less than 1%

Acc: accuracy; AE: absolute error; BPF: bandpass filter; BSS: blind source separation; CNN: convolutional neural network; CWT: continuous wavelet transform; Dev.: device; DWT: discrete wavelet transform; EMD: empirical mode decomposition; FIR: finite impulse response; Gest.: gestational; HMM: hidden Markov model; HPF: high-pass filter; HRV: heart rate variability; IIR: infinite impulse response; IUP: intrauterine pressure; LMS: least mean squares; LPF: low-pass filter; N: no; NNMF: non-negative matrix factorization; Pop.: population; PSD: power spectral density; R: correlation coefficient; Ref.: reference; RLS: recursive least squares; SD: standard deviation; SNR: signal-to-noise ratio; SVR: support vector regression; Y: yes.

## 4. Discussion

The goal of this section is to discuss the results presented in Section 3. The discussion will focus on the general characteristics of the device, on the system architecture and the sensors, and on the signal processing and validation performed. A comparison against other state-of-the-art reviews will be proposed. In the end, the open challenges will be summarized along with the limitations of the study.

### 4.1. General Overview

This scoping review (i.e., a type of knowledge synthesis that uses a systematic and iterative approach to identify and synthesize an existing or emerging body of literature on a given topic [78]) investigated the research prototypes (RPs) and commercial devices (CDs) for the acquisition of FPCG in the field of fetal monitoring. Indeed, the topic of devices related to FPCG recording is a historical and large theme, never deeply reviewed and synthesized in the literature. Indeed, the first research publication related to FPCG acquisition dates to 1941, and the research is still active on the topic. Thus, we believe that a comprehensive review of all these devices may be useful for future industrial research on the topic, summarizing the innovations already presented in the literature and raising the challenges that are still open.

By considering the obtained results, the selected 57 studies were published from 1941 to 2023. Despite journal papers being the most present, the number of conference contributions (44%) is similarly high. This evidence is probably related to the fact that the research devoted to design, prototype, and validating a new device requires more time than research devoted to clinical applications and software development. Thus, authors usually present preliminary results of their activity before conferences (by considering uniquely a qualitative validation) to obtain feedback and optimize their work. Then, after prototyping and validation, the entire work is usually published in a journal.

Overall, the selected 57 studies described 34 devices for FPCG acquisition. As highlighted in Section 2.3, these devices may be classified as an RP or CD (Figure 4A). Of note, RPs (26) are more numerous than CDs (8), and this fact reflects the normal pipeline of device commercialization. Indeed, CDs required multiple setting adjustments and optimization, and several clinical validations before being commercialized in the market. Moreover, few RPs evolve into new CDs, but their industrial innovation may be integrated into an already commercialized CD.

About the time evolution of the technology related to FPCG acquisition (Figure 4B,C; note that the last class includes only 4 years), it is obvious that the first devices, from 1941 to 1980, were analog. Indeed, the unique invasive device [25], whose description was published in 1964, was analog. In the 1990s, analog devices started to be substituted by digital devices. In Figure 4B, it is notable that the research was very active between 2000 and 2009. Then, the research started to be converted into commercialization, considering the increasing CDs. Specifically, while the research activity was focused on all types of devices, the commercialization involved principally SC and MS devices.

Finally, the analysis of Table 2 and Table 3 highlights that the research on the FPCG device seems to consider a combined MC and MS approach, without considering the two types separately. This is interesting because it is in line with the recent industrial research on wearable sensors that integrates different types of sensors and different leads of the same system to collect more information in a unique acquisition. Moreover, considering that most of the MS devices acquired also maternal signals, this approach proposes to consider mother and fetus as a unique physiological system, differently from the current clinical practice.

### 4.2. System Architecture and Use of Sensors

The sensing front-end is a fundamental aspect of every device for the monitoring of noninvasive biomarkers. On one hand, it influences the wearability and the usability of the device, which is an important characteristic of monitoring devices, as highlighted in the Introduction. This is particularly relevant when the device is devised for use in telemonitoring applications, where the patient or a caregiver is in charge of recording signals of sufficient quality. On the other hand, the sensing front-end represents the first stage of the monitoring process: no reliable information can be extracted from badly recorded signals or from missing signals.

**Figure 7:**
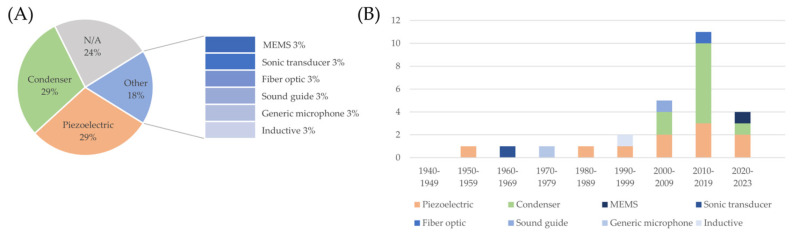
Distribution of the kind of microphone used in the devices described in the included studies (panel (**A**)) and trend over the decades (panel (**B**)).

The front-end grounds on the sensors. The focus of this work is on heart sounds; therefore, a microphone is an obvious common characteristic of all the devices. It should be highlighted that similar information can be extracted from accelerometers or other vibration sensors, but the latter fall outside the scope of this review. The choice for a suitable type of microphone is not straightforward, given that the bandwidth of fetal heart sounds is in the low-frequency spectrum, whereas common microphones are typically optimized for higher frequencies, and it is technically difficult to cover low frequencies without increasing the size of the sensor. The analysis of the type of microphone sensors employed in the devices summarizes the information from Table 2 and Table 3 and is reported in Figure 7. Two microphone types emerged as the most typical choice, i.e., piezoelectric microphones and condenser microphones (electret in almost all cases). In particular, the use of piezoelectric sensors was quite stable over the years, and still common to date, while condenser microphones took over starting from 2000. It is important to highlight that solutions based on other types of sensors have been explored throughout the timeline, showing that there is still room for innovation.

**Figure 8:**
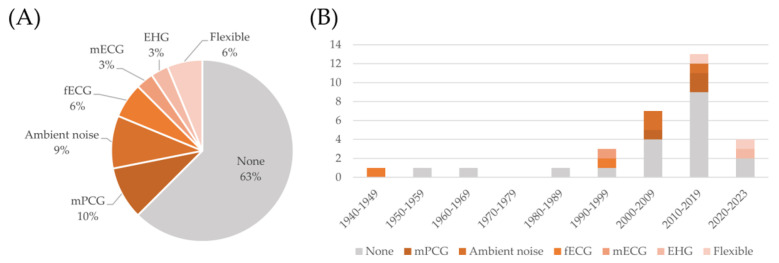
Distribution of the kind of signals acquired by the devices described in the included studies (panel (**A**)) and trend over the decades (panel (**B**)).

As discussed in Section 4.1, the use of multimodal systems, involving the simultaneous recording of multiple signals, has been an object of research in the latest decades. The use of multiple sensors has, according to the analyzed sources, two main goals. The first is to improve the quality of the FPCG signal, which is still the main target of the monitoring. This can be obtained by recording multiple channels and by using blind source separation techniques, or by recording potential sources of noise, such as ambient noise or the maternal PCG, to be removed from the signal of interest. The second goal is to add complementary information by recording simultaneous signals of interest, such as the fetal electrocardiogram (FECG), the maternal ECG, or the uterine electrical activation, i.e., the electrohysterogram. Some commercial platforms provide more flexibility in selecting and connecting different types of sensors to the data acquisition unit. Figure 8 reports the distribution of the use of these signals in the analyzed devices.

**Figure 9:**
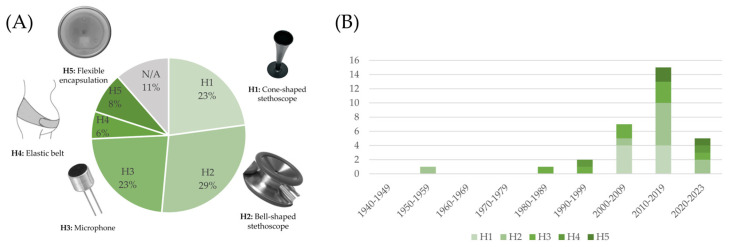
Distribution of the type of head employed in the devices described in the included studies (panel (**A**)) and trend over the decades (panel (**B**)).

It should be noted that single-sensor systems are still predominant to date. These devices are typically based on a single microphone and are designed to acquire an FPCG signal alone. In other words, the mentioned devices are basically electronic stethoscopes optimized for use on the maternal abdomen for fetal monitoring purposes. This is reflected in the type of head, described in Section 3 and Figure 5. As highlighted in Figure 9 (panel A), more than half of the studies, either using a self-designed RP or a CD, ground the sensing front-end on a stethoscope head, either adapted from an existing stethoscope or purposely developed.

This is especially evident in studies using a CD: as highlighted in Table 3, in all cases, with a single exception, the employed device is a generic electronic stethoscope rather than a fetal-specific device. The classical stethoscope head (either in its cone-shaped or in its bell-shaped form) is known to provide an optimal conduction of the acoustic waves from human skin to the sensor. Nevertheless, when multiple sensors (microphones or other types) come into the picture, the situation changes: embedding multiple sensors into the same stethoscope head is complex and most often suboptimal (different positions are required for different sensors), and using each sensor separately hugely complicates the system. Moreover, the use of a common electronic stethoscope has an impact on the usability of the device in a domicile setting for telemonitoring applications. In fact, it is well known that trained clinical staff are required to perform fetal auscultation with a traditional or an electrical stethoscope.

A few works have explored the use of more complex front-ends, either using a belt embedding the microphone (and possibly other sensors) or designing a custom flexible encapsulation. The advantage of similar technological solutions is two-sided: on one side, the relative position of the sensors on the maternal abdomen is fixed, enabling an easier positioning by inexperienced users; on the other side, the use of a belt or a similar fastening means avoids the user having to hold the stethoscope while performing the recording, which is a common source of artifacts. Figure 9 (panel B) shows that the use of a flexible encapsulation embedding the microphone and the other sensors increased in the latest decade, highlighting that researchers are striving to find novel technological solutions to make acoustical fetal monitoring feasible in a telemedicine context.

**Figure 10:**
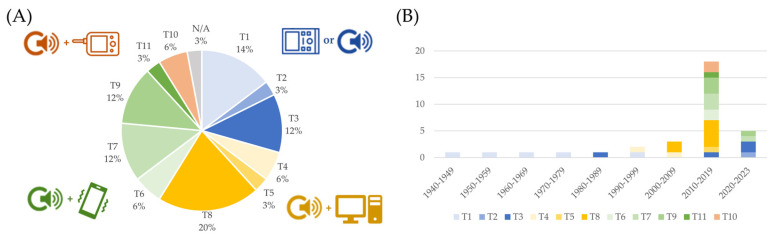
Distribution of the system architecture described in the included studies (panel (**A**)) and trend over the decades (panel (**B**)). Blue represents stand-alone systems or studies describing the sensing front-end alone, yellow represents computer-based systems, green represents smartphone-based systems, and red represents systems based on a commercial portable monitor.

A further topic of interest is related to the architecture of the system, described in Section 3 and Figure 6. Figure 10 reports on the distribution of identified patterns, as defined in Figure 6.

Stand-alone, computer-based, smartphone-based, and monitor-based systems are highlighted. Systems developed before 1990 are mostly analog, as discussed in Section 4.1: they are mostly stand-alone devices, which embed the sensor front-end, the electronical circuits, and the data acquisition module (when available, most devices were analog) in the same apparatus, which also provides the user with direct feedback on the status of the fetus. The evolution of the system architecture in the following years reflects the evolution of the telecommunication technology. In fact, in the 2000–2009 decade, computer-based systems were the standard; smartphone-based systems emerged in 2010 and were equally chosen in the following decade; smartphone-based systems were mostly employed from 2020 on. The two technologies could probably coexist in the future because they target two different needs: computer-based systems can be reliably employed in ambulatory assessments, whereas smartphone-based systems are the best choices for telemonitoring. Stand-alone systems emerged again in the latest years, possibly connected to a cloud for computing and data storage purposes. This new direction may be further explored in the future.

**Figure 11:**
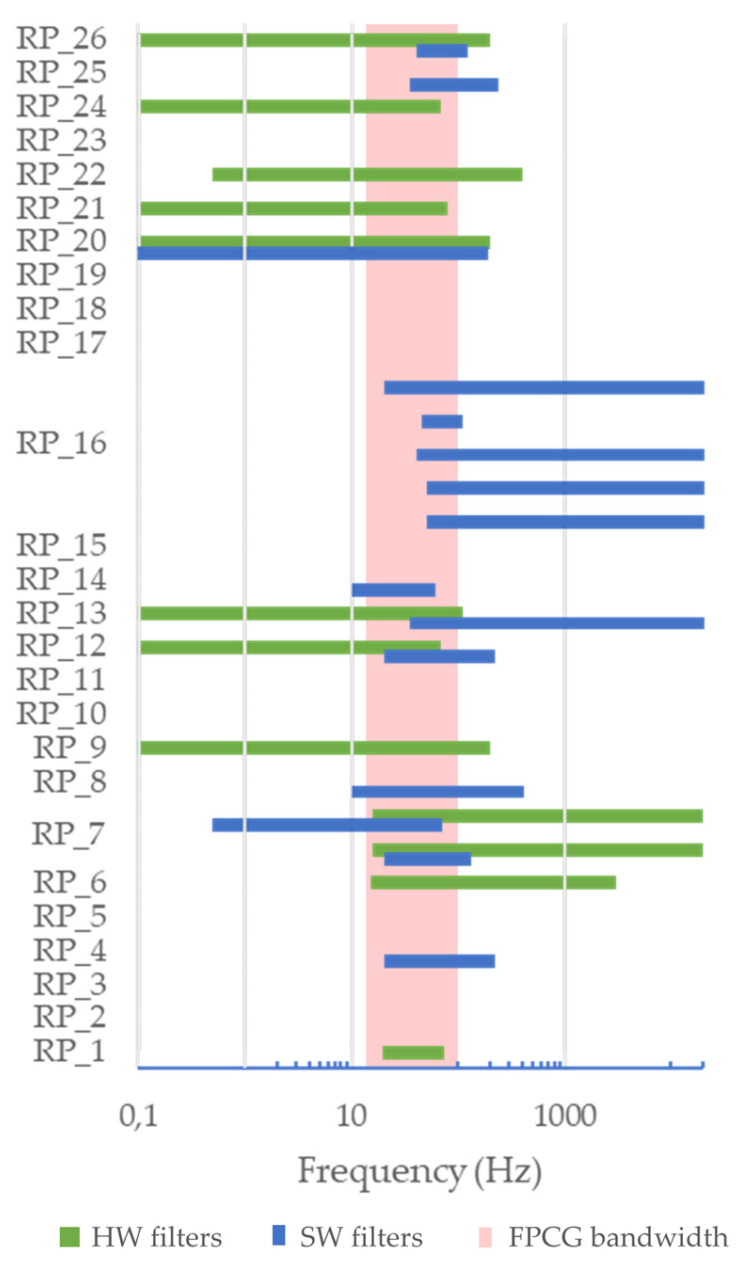
Frequency bandwidth of the hardware (in blue) and software (in green) filters, respectively, compared with the bandwidth of FPCG.

Concerning hardware implementation, it is definitely an underinvestigated aspect in the literature as we can draw from the reported results (Table 2 and Table 3). In fact, most studies, even if aimed at describing a novel RP, mentioned few details about the actual hardware specifications. This limits the reproducibility of the research. The most reported information is the sampling frequency, along with the dynamics of the Analog-to-Digital Converter (ADC) and the frequency limits of the filters. The sampling frequency ranges from 640 Hz to 250 kHz (median: 3.5 kHz). It should be highlighted that in some systems the recording is performed at high frequency, but then down-sampling is performed in the processing phase. A sampling frequency of a few kilohertz is sufficient to obtain a reliable estimation of the FHR and its variations. The dynamics of the ADC was 16 bits in most cases. The frequency bandwidth of the hardware filters is reported in Figure 11 for each study. It can be noted that in studies where both the hardware and the software filters are described, the bandwidth of the hardware filter typically spans wider frequencies. The typical bandwidth of the fetal heart sounds is often obtained as the intersection between the bandwidth of the hardware and software filters (when both available). It can also be noted that the agreement among different RPs is low: a better understanding of the frequency content of fetal heart sounds may provide an improvement in this sense in the future.

### 4.3. Signal Processing

Each of the devices discussed in Section 4.2 can work independently of the type of processing that will be performed on the signals. Nevertheless, recording the fetal heart sounds is meaningful only if relevant information for the monitoring of the fetal health status can be extracted. This is achieved by means of signal processing.

For the analysis of signal processing features, the studies were analyzed separately, even if they rely on the same recording system: in fact, different studies may use the same device but update the signal processing methodology over the time to improve the performances. As noticeable from Table 4, most of the studies (33 out of 57) in this study cohort use the device for FHR monitoring. This is achieved by means of two processing steps, namely denoising and FHR estimation. Denoising methods include the following:

1. Digital filtering (25 studies). A detailed analysis of the reported frequency bandwidth is proposed in Figure 11. As already discussed in Section 4.2, the use of digital filtering is typically devoted to limiting the bandwidth of the signal to the typical bandwidth of the two main fetal heart sounds (20 Hz to 120 Hz);
2. Wavelet decomposition (nine studies);
3. Adaptive filtering (four studies);
4. Other methods including support vector regression (two studies), empirical mode decomposition (two studies), computational auditory scene analysis (one study) and adaptive cross-channel cancellation (one study).

**Figure 12:**
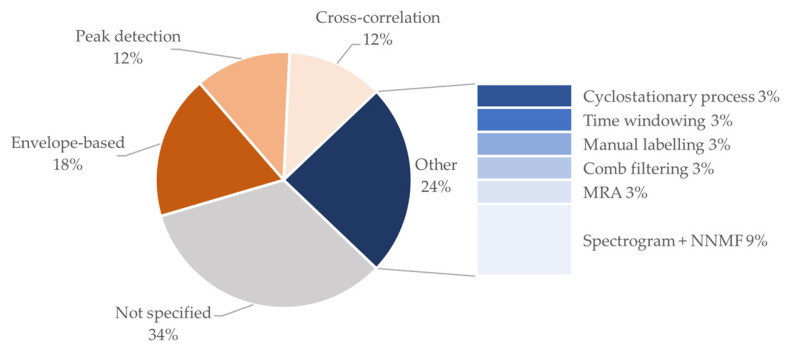
Proportion of studies using various signal processing methods. MRA: multi-resolution analysis. NNMF: non-negative matrix factorization.

It should be highlighted that a non-negligible number of the included studies reports the use of multiple denoising methods in combination (typically digital filtering followed by another of the mentioned methods). For the estimation of the FHR, three main methods are reported, namely envelope-based estimation, peak detection, and cross-correlation. Figure 12 shows a detailed distribution of the explored methods.

It can be noted that most works report the use of simple signal processing methods for FHR estimation. Even though more complex approaches exist in the literature, this should be viewed in light of the fact that the main goal of the included studies is the validation of the device and not the improvement of the FHR estimation from the signal.

Therefore, established methods are typically preferred. A non-negligible percentage of the studies in the study cohort (34%) reports FHR results without specifying the signal processing method. The latest result shows that often in the literature device validation and signal processing are still considered separated, even if the second strongly influences the validation performances of the first.

In addition to FHR estimation, which was found to be the most common processing goal, the following types of analysis were carried out and reported in Table 4:

- Separation of fetal and maternal heart sounds by means of adaptive filtering and least mean square (five studies) or blind source separation (two studies);
- Segmentation of the first and second heart sounds by means of envelope-based peak detection and cardiac time intervals estimation (four studies);
- Power spectrum analysis (three studies);
- Time-frequency analysis using spectrograms (one study);
- Identification of fetal movements by means of filtering (one study);
- Heart rate variability estimation and analysis (one study);
- Single-channel blind source separation using a combination of empirical mode decomposition and non-negative matrix factorization (one study);
- Fetal heart localization using convolutional neural networks on images built on top of the power spectrum (one study)
- Confidence factor estimation (one study)

In this case, the validation was often difficult to perform as no gold standard exists. Nevertheless, the number of different signal processing tasks performed on the recorded signal in addition to FHR estimation shows that even if the use of FPCG for fetal monitoring was first introduced for FHR, its potentiality goes well beyond. In fact, as highlighted in the Introduction, FPCG reflects multiple aspects of the behavior of the fetal heart and can be used for a full fetal heart assessment, bringing the follow-up visit to the domicile of the pregnant woman.

### 4.4. Validation

Similarly to signal processing, validation is a core part of the design and development of a device because it is focused on the demonstration of its utility and clinical relevance. Despite some studies describing the same device, the studies reported different clinical evaluations. Thus, Table 4 was stratified according to the studies and the related device.

In the study cohort, four studies did not describe a validation, causing missing information of all validation fields in Table 4. By considering the devices of these studies, three out of four refer to RP: information regarding the validation of two studies is presented in other studies that referred to the same RP, while for the remaining RP, validation is not present. Finally, the fourth device is a CD, and its study presented no validation information.

**Figure 13:**
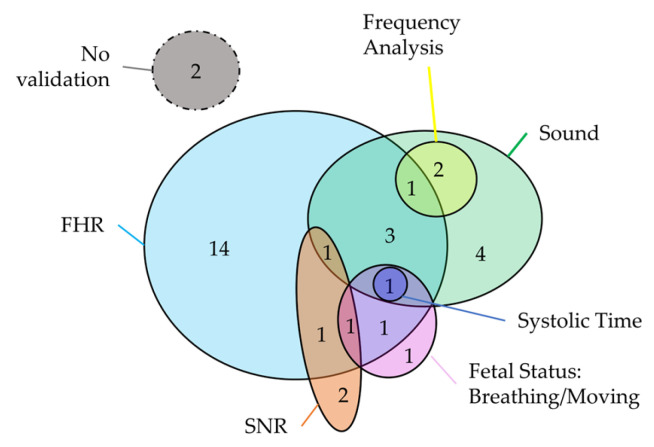
Venn diagram of the combination of the different clinical and technical features used for devices validation.

Most of the studies aimed at clinically evaluating the device (32 studies), which consists of the assessment of FPCG acquisition by using clinical features. Specifically, the considered clinical features are related to FHR and its variability, fetal status (fetus alive, breathing, and moving) and systolic time. The use of FHR and its variability is perfectly in line with the clinical practice, considering that the FHR is the main index for the assessment of fetal wellbeing according to the main clinical guideline [7,8,9,10]. Of note, two studies evaluated the sound generated by fetal movement, which is a secondary index of fetal wellbeing. Thus, this validation evidence underlines the advantage of FPCG that can be used for the estimation of multiple indexes of fetal health status, as highlighted in the Introduction. Moreover, 19 studies aimed to technically evaluate the device by computing the quality of the sound, SNR of the acquired FPCG, and performing a frequency analysis. Finally, three studies presented a dual validation by considering both clinical and technical features. Regarding the devices, two devices were not validated, sixteen devices were only clinically validated, eight devices were only technically validated, and eight devices were clinically and technically validated. The Venn diagram in Figure 13 represents the combination of the different clinical and technical features that aimed to validate the devices.

Clinical validation results are the most important, considering that that represents the applicability of the acquisition system in a real clinical scenario. On the contrary, the unique technical validation may be not sufficient for the clinical use of the device.

**Figure 14:**
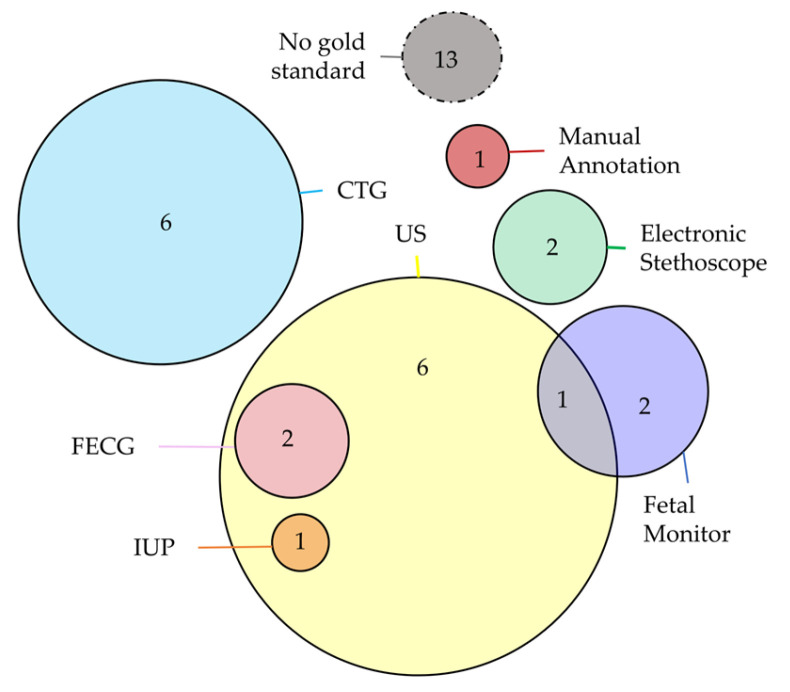
Venn diagram of the combination of the different technologies that act as gold standard in the device validation procedure.

Device validation is usually performed by considering a simultaneous reference (gold standard) acquisition system. Among the selected studies, 29 studies did not present a comparison with a reference system but considered only the single measure of the proposed device. On the other hand, twenty studies compared their results with clinical instrumentation: specifically, eleven studies considered the CTG, and nine studies considered FECHO, which are the most common methodologies used in the clinical practice, as presented in the Introduction. About the other ones, these studies considered alternative measurements, such as fetal monitor (four studies), FECG (two studies), electronic stethoscope (two studies), and manual annotations (one study). Finally, two studies considered invasive methods, such as the intrauterine pressure catheter (IUP) or the scalp FECG. Despite two studies using the stethoscope, none of the studies validated the quality of the signal by directly comparing the signal with those acquired by another FPCG device. This setting choice would help to directly validate the morphology of the signal, without passing through a feature extraction procedure that can be corrupted by the used signal processing algorithm. The Venn diagram in Figure 14 represents the combination of the different technologies that act as the gold standard in the device validation procedure. The validation was performed on a population size ranging from 1 pregnant woman to 140 pregnant women, with a distribution of 20 ± 27 pregnant women. Moreover, 17 studies did not report the information regarding the used population. Considering the clinical features of these populations, the gestational age ranges from 13 weeks to 42 weeks.

**Figure 15:**
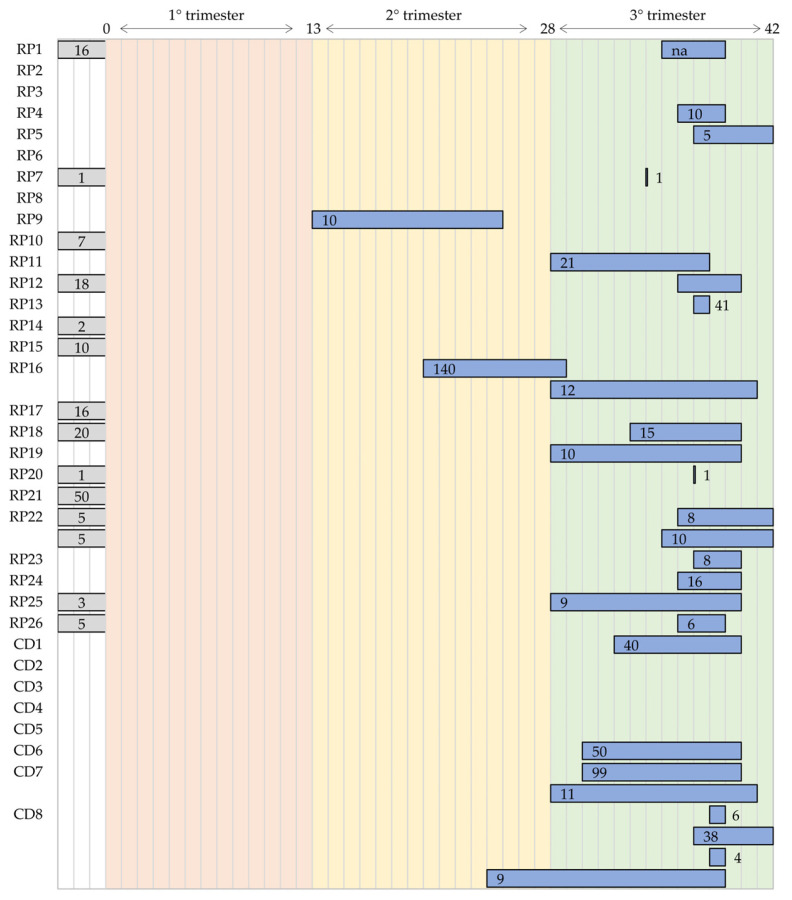
Distributions of the population used in the clinical validation of the research prototypes (RPs) and commercial devices (CDs) in the entire pregnancy period.

Figure 15 represents the distributions of the validation population during the pregnancy period. Of note, RP22, CD7, and CD8 [55,56,57,58,59,70,72,73,74,75,76,77] were validated in more than one population. Moreover, for 13 devices, the validation is present, but the information related to the gestational age is missing (gray bar in Figure 15). By considering the results, the studies mainly focused on the third trimester, and some of them considered the second trimester. No studies evaluated the performance of the devices during the first trimester, despite the physiological evidence that the fetal heart starts to beat within the sixth week. Moreover, none of the studies detailed the clinical situation of both mother and fetus. This information would be very helpful to understand the applicability of the devices in different contexts, such as in mothers affected by preeclampsia or gestational diabetes or in fetuses affected by hypoxia or intrauterine restriction growth.

By considering the clinical validation results, most of the studies (11 studies) applied a qualitative appraisal, without applying a rigorous statistical analysis. About the quantitative assessment, most of the studies performed an SNR evaluation and differences in terms of FHR. Correlation analysis, Bland–Altman plot, absolute error, and accuracy computation are also presented. All the studies reported excellent results in terms of statistical analysis. The most interesting clinical evaluations are those of RP22 [55,56,57,58,59] that used simulated data for quality assessment and those of RP16 [43,44,45,46,47] that stratified the results according to the gestational age. These results open the idea of new approaches for the FPCG quality assessment. Indeed, hardware simulators may help the validation of the device not only during the clinical stages but also during the design and development phases. Moreover, the evidence observed in the different gestational ages may suggest the adjustment of the device setting according to the gestational age of the woman.

### 4.5. Comparison against State-of-the-Art Reviews

During the scoping review search activity, 26 papers were excluded as review papers. Most of these studies reviewed the literature by considering the clinical guidelines for the interpretation of FPCG. By considering the scope of our review, we decided to exclude these papers by considering the defined eligibility criteria. Among them, two focused on the FPCG recording and on its use for fetal wellbeing assessment [19,20]; thus, we considered these two studies as competitors in the state of the art (Table 5).

The first review was published by Kovács et al. [19] in 2011. This review included the general physiological description of FPCG by considering the genesis of phonocardiography, the modeling of fetal heart sounds, and the role of FPCG for a fetal distress diagnosis. Thus, despite the presence of a detailed description of the signal of interest, the core of the review is not focused on the details of the acquisition procedure. The second review was published by Adithya et al. [20] in 2017. Also, in this case, the physiological aspects related to FPCG were introduced, together with a detailed description of the interferences that may affect the recording. Moreover, Adithya et al. [20] mainly focused on the signal processing analysis related to FPCG. This finding is perfectly in line with the entire literature on the topic: indeed, most of the papers published in the 2010s (excluded by our eligibility criteria) focused on the development of new algorithms and technologies to process the FPCG, independently of the acquisition setting.

Both reviews introduced issues related to the FPCG acquisition by introducing the importance of sensor settings (sensing device and probe location), integration of filtering procedure on the device, and the importance of the clinical validation (specifically in the case of pathology). Despite these topics being treated, a comprehensive and systematic overview of the literature on the hardware requirements was not presented. Thus, in comparison with the state of the art, our scoping review is the first and only one that has performed a detailed analysis of RPs and CDs that aimed to acquire FPCG, with the specific objective to use the FPCG for fetal home-monitoring.

**Table 5:** Reviews comparison.

Ref.	Year	Focus	Type/ Guideline	FPCG Physiology and Modeling	FPCG Acquisition Systems	Signal Processing	Clinical Validation
Kovács et al. [19]	2011	Overview of FPCG works on the applied signal processing methods for identification of sound components	-	YES	Briefly introduced	Generally treated	Generally treated
Adithya et al. [20]	2017	Trends in data collection, signal processing techniques, and synthesis models related to FPCG	-	YES	Briefly introduced	Systematically analyzed	Related to signal processing
Proposed review	2024	Investigation and trends of the FPCG acquisition systems	Scoping/ PRISMA	NO	Systematically analyzed	Analyzed in connection to the acquisition system	Related to acquisition system

### 4.6. Open Challenges

Considering the results of this review, some challenges are still open. To our knowledge, as discussed in Section 4.1, no CD was explicitly designed for fetal applications, which means that used devices are those developed for adult phonocardiogram acquisition. This negatively impacts on the usability of the devices that need to be used only by clinical personnel, who have to find the best auscultation site. Consequently, the actual technology is not transferable to the wearable device/in-home monitoring market. Indeed, our literature review did not find any fetal-specific CDs as used devices or gold standard devices. If such CDs exist, they are not included in the standard research activities and thus are not used to validate the novel RPs. If they do not exist, industrial research is still open to innovation.

About the decision settings on multiple channels and multiple sensors, research is going in the direction of multimodality, as discussed in Section 4.2. These new multi-sensor and multi-lead configurations involve new requirements in terms of the shape and fastening of the wearable front-end, in particular for telemonitoring applications, to ensure the usability of the device by inexperienced users and comfort for the patient. Then, new multi-lead configurations will require a specifically designed algorithm for the selection of the optimal lead of auscultation, considering the non-standardized position of the fetus with respect to the maternal abdomen. Moreover, the use of multimodality may consider the standard guidelines for fetal monitoring, embedding both the FHR estimation module and the maternal UC module, to provide a complete monitoring of the pregnancy. The possibility of extracting multiple biosignals with the same devices also opens the possibility to design new indexes of fetal wellbeing (such as fetal movement indexes), providing more precise and more robust monitoring possibilities with respect to state-of-the-art technology for fetal monitoring. System architecture can be devised according to two main directions, which are computer-based systems mainly used for ambulatory and by clinical personnel, and smartphone-based systems, used by the patient during her routine activity.

As highlighted in Section 4.3, most of the open challenges are related to the validation phase. Indeed, the observed validation procedures are typically focused on the last trimester of gestation. Nevertheless, the heart of the fetus starts beating during the first trimester, specifically during the sixth week of gestation. Thus, testing the devices on previous gestational weeks may provide deeper insight into fetal heart development and may open up new monitoring possibilities in the future. Then, the validation procedures are usually performed on healthy pregnancies (both from the mother and the fetus perspective), without considering the clinical outcomes of the delivery or the possibility of a multiple pregnancy. Of note, only one of the found devices (RP20) was tested in the case of multiple pregnancy [51,52]. Thus, in the future, it will be interesting to explore the limits of this technology when considering extreme FHR (such as bradycardia, tachycardia, or other arrhythmias), the presence of fetal (such as hypoxia or intrauterine growth retardation) or maternal (such as preeclampsia or gestational diabetes) pathologies, the presence of a correlation with adverse clinical outcomes and in the presence of a multiple pregnancy. Finally, all validation procedures are based on quality appraisal or on feature extraction; thus, a technical comparison with a simultaneously acquired FPCG waveforms is desirable in the future.

### 4.7. Limitations

Despite our belief in the innovation and utility of this review, it is not free of limitations. Many presented papers do not fully describe the hardware implementation, thus preventing, to some extent, the reproducibility of their device. Moreover, many works do not perform complete clinical validation against a recognized gold standard or do not provide any kind of quantitative validation. Therefore, the device is to be considered validated from a technical perspective but not for the purposes of the clinical application of interest, at least according to the included studies. Considering this evidence, some devices presented missing information, which, consequently, are missing also in this scoping review. Finally, observing the complexity of the devices and system architectures, an estimation of the costs for the commercialization of these devices cannot be performed, which would be of interest in terms of industrial research activity.

## 5. Conclusions

This scoping review systematically explored the technological solutions devoted to recording fetal heart sounds and highlighting the trends and innovations. From our review study, it emerged that commercial devices are not fetal-specific, and most studies strive to overcome the issue by proposing novel research prototypes: in this sense, the field is still open to innovation. While the first prototypes basically emulated the electronic stethoscope, the latest advances involve the use of multiple microphones and multiple sensors.

Future research directions may focus on novel technology and clinical validation of acquired signals in a real-life scenario. Future technology is expected to ground on multimodality (with a specific focus on the combination of FHR extraction and UC acquisition) to bring fetal monitoring to the patient’s domicile and to provide a complete overview of the health status of the mother–fetus system. Quantitative validation of the novel proposed research prototypes is crucial for bringing the device into the clinical pathways. In the future, validation should include an FPCG gold standard and not rely on extracted features whose goodness may be impacted by the signal processing procedure. In the end, future systems should integrate both the hardware and the software components to bridge the gap between recording and interpretability, enabling fetal monitoring in a telemedicine framework.

## Figures and Tables

**Table 1:** Details about the searched databases, search parameters, and search query.

Database	Search Parameters	Query	Accessed on
Scopus	Article title, abstract, keywords	(“Fetal” OR “Pregnancy” OR “Fetus” OR “Prenatal” OR “Antenatal” OR “Foetal” OR “Foetus”) **AND** (“Phonocardiography” OR “Heart Sound” OR “FPCG” OR “PCG” OR “Heart Murmur” OR “Acoustic cardiography” OR “Auscultation”) **AND** (“Hardware” OR “Device” OR “System” OR “Recording” OR “Acquisition” OR “Microphone”)	2 November 2023
PubMed	All fields		2 November 2023
Web of Science	Title, abstract, Keyword Plus ^®^		2 November 2023
